# Sorafenib analogue SC‐60 induces apoptosis through the SHP‐1/STAT3 pathway and enhances docetaxel cytotoxicity in triple‐negative breast cancer cells

**DOI:** 10.1002/1878-0261.12033

**Published:** 2017-02-07

**Authors:** Chun‐Yu Liu, Jung‐Chen Su, Tzu‐Ting Huang, Pei‐Yi Chu, Chun‐Teng Huang, Wan‐Lun Wang, Chia‐Han Lee, Ka‐Yi Lau, Wen‐Chun Tsai, Hsiu‐Ping Yang, Chung‐Wai Shiau, Ling‐Ming Tseng, Kuen‐Feng Chen

**Affiliations:** ^1^ Comprehensive Breast Health Center Taipei Veterans General Hospital Taiwan; ^2^ Division of Medical Oncology Department of Oncology Taipei Veterans General Hospital Taiwan; ^3^ School of Medicine National Yang‐Ming University Taipei Taiwan; ^4^ Institute of Biopharmaceutical Sciences National Yang‐Ming University Taipei Taiwan; ^5^ Department of Clinical Laboratory Sciences and Medical Biotechnology National Taiwan University Taipei Taiwan; ^6^ Department of Pathology Show Chwan Memorial Hospital Changhua Taiwan; ^7^ School of Medicine College of Medicine Fu‐Jen Catholic University Xinzhuang New Taipei City Taiwan; ^8^ Division of Hematology & Oncology Department of Medicine Yang‐Ming Branch of Taipei City Hospital Taiwan; ^9^ Department of Surgery Taipei Veterans General Hospital Taiwan; ^10^ Department of Medical Research National Taiwan University Hospital Taipei Taiwan; ^11^ National Taiwan University College of Medicine Taipei Taiwan

**Keywords:** SHP‐1 agonist, STAT3, triple‐negative breast cancer

## Abstract

Recurrent triple‐negative breast cancer (TNBC) needs new therapeutic targets. Src homology region 2 domain‐containing phosphatase‐1 (SHP‐1) can act as a tumor suppressor by dephosphorylating oncogenic kinases. One major target of SHP‐1 is STAT3, which is highly activated in TNBC. In this study, we tested a sorafenib analogue SC‐60, which lacks angiokinase inhibition activity, but acts as a SHP‐1 agonist, in TNBC cells. SC‐60 inhibited proliferation and induced apoptosis by dephosphorylating STAT3 in both a dose‐ and time‐dependent manner in TNBC cells (MDA‐MB‐231, MDA‐MB‐468, and HCC1937). By contrast, ectopic expression of STAT3 rescued the anticancer effect induced by SC‐60. SC‐60 also increased the SHP‐1 activity, but this effect was inhibited when the N‐SH2 domain (DN1) was deleted or with SHP‐1 point mutation (D61A), implying that SHP‐1 is the major target of SC‐60 in TNBC. The use of SC‐60 in combination with docetaxel synergized the anticancer effect induced by SC‐60 through the SHP‐1/STAT3 pathway in TNBC cells. Importantly, SC‐60 also displayed a significant antitumor effect in an MDA‐MB‐468 xenograft model by modulating the SHP‐1/STAT3 axis, indicating the anticancer potential of SC‐60 in TNBC treatment. Targeting SHP‐1/p‐STAT3 and the potential combination of SHP‐1 agonist with chemotherapeutic docetaxel is a feasible therapeutic strategy for TNBC.

**Abbreviations**
SHP‐1: src homology region 2 domain‐containing phosphatase‐1
STAT3: signal transducer and activator of transcription 3
TNBC: triple‐negative breast cancer

## 1 Introduction

Triple‐negative breast cancer (TNBC), a heterogeneous breast cancer subtype, is known for its poor prognosis and high rate of metastasis. In addition, a considerable proportion of patients with TNBC are also associated with BRCA gene mutation (Lehmann *et al*., 2011). Despite the discovery of the poly (ADP‐ribose) polymerase (PARP) inhibitors that interfere with DNA damage repair and their implications in BRCA‐deficient TNBCs, currently there are no approved targeted therapies for TNBC (Mayer *et al*., 2014).

Signal transducer and activator of transcription 3 (STAT3) mediates a plethora of cellular functions in response to cell stimuli by cytokines (such as interleukin 6; Darnell *et al*., 1994) and growth factors (such as epidermal growth factor; Song and Grandis, 2000). Upon phosphorylation at tyrosine 705 residue, STAT3 transcriptionally regulated genes involved in cell growth, division, cell movement, apoptosis, and so on (Fukada *et al*., 1996; Kalluri, 2003; Zhang *et al*., 2005). In cancers, including TNBC, STAT3 is constitutively activated and frequently associated with poor prognosis and tumor resistance to anticancer therapy (Banerjee and Resat, 2015; D'Anello *et al*., 2010; Marotta *et al*., 2011; Real *et al*., 2002; Wei *et al*., 2014). The tumor resistance to anticancer therapy can be attributed to the participation of STAT3 in antiapoptosis by upregulating antiapoptotic proteins (bcl‐2, Mcl‐1, survivin, etc.; Banerjee and Resat, 2015; Berishaj *et al*., 2007; Diaz *et al*., 2006; Gritsko *et al*., 2006; Hartman *et al*., 2013) or by activating cell cycle mediators (such as cyclin D1) and many other STAT3‐regulated genes involved in prosurvival signaling and self‐renewal of cancer stem cells (Rajendran *et al*., 2012; Tan *et al*., 2014; Yao *et al*., 2011). Increased STAT3 activity has also been linked to the development of chemoresistance in TNBC (Gariboldi *et al*., 2007), as well as associated with metastasis promotion in TNBC (Lee *et al*., 2011). Moreover, newly identified cancer‐promoting functions of STAT3 – its role in mitochondria, epigenetic regulation, cancer stem cells, obesity, and premetastatic niches – further highlight the importance of targeting STAT3 in cancers (Yu *et al*., 2014). Taken together, these findings suggest that targeting STAT3 has therapeutic potential and might offer clinical benefits for patients with TNBC.

A number of agents and natural compounds have been reported to inhibit STAT3 using various strategies (Chai *et al*., 2016; Furtek *et al*., 2016). Common STAT3‐targeting approaches include inhibiting upstream tyrosine kinases that phosphorylate/activate STAT3 (such as JAKs), and small molecules blocking functional STAT3 dimerization via interfering with the SH2 domains of STAT3 (Furtek *et al*., 2016). Notably, ruxolitinib, a JAK1/2 tyrosine kinase inhibitor, is currently being tested in phase II trials for solid tumors such as HER‐2‐negative metastatic breast cancers (NCT02120417) and pancreatic cancers (Hurwitz *et al*., 2014). In addition, compounds suppressing STAT3 phosphorylation independently of JAK inhibition are also attracting attention. Another emerging strategy targeting is the negative regulation of STAT3 signals (Fan *et al*., 2014; Liu *et al*., 2013, 2014; Tai *et al*., 2011, 2014a,b). Src homology region 2 domain‐containing phosphatase 1 (SHP‐1), a nonreceptor protein tyrosine phosphatase, is one of the negative regulators of phosphorylated STAT3 (p‐STAT3; Lopez‐Ruiz *et al*., 2011). SHP‐1 is composed of two SH‐2 domains (N‐SH2 and C‐SH2) and one catalytic PTP domain (Yang *et al*., 1998). Studies have shown that the activity of SHP‐1 is also regulated by the conformational rearrangement upon substrate/chemical binding: The N‐SH2 domain is released from the interaction with the PTP domain exposing the catalytic site of the PTP domain, thereby enhancing SHP‐1 activity (Qin *et al*., 2005; Wang *et al*., 2011; Yang *et al*., 2003). Interestingly, we identified that multiangiokinase inhibitor sorafenib also acts as a direct enhancer of SHP‐1 (Tai *et al*., 2011, 2014b). Accordingly, we developed a series of sorafenib derivatives that are devoid of angiokinase (VEGFR/PDGFR) inhibition and demonstrated that they inhibited p‐STAT3 via increasing SHP‐1 activity (Chen *et al*., 2011, 2012c; Su *et al*., 2012). We previously demonstrated the *in vitro* and *in vivo* anticancer activity of a SHP‐1 agonist, SC‐43, in TNBC cells (Liu *et al*., 2013). More recently, we showed a chemical dimeric sorafenib derivative, SC‐60, with survival benefits compared with sorafenib in a hepatocellular carcinoma orthotopic model (Fan *et al*., 2014). In the current study, we report the effects of SC‐60 in TNBC cells.

## 2 Materials and methods

### 2.1 Reagents and antibodies

SC‐60 was synthesized by Hinova Pharmaceuticals Inc. (Chengdu, China), the MW of SC‐60 is 473, and its structure and solubility are described in Fig. S1. For *in vitro* studies, SC‐60 at various concentrations was dissolved in dimethyl sulfoxide (DMSO) and added to cells in Dulbecco's modified Eagle's medium (Invitrogen, Carlsbad, CA, USA). The final DMSO concentration was 0.1% after addition to the medium. For *in vivo* studies, SC‐60 was dissolved in 50% (v/v) propylene glycol and 50% (v/v) Solutol^®^ HS‐15. The SHP‐1 inhibitor PTP inhibitor III (CAS 29936‐81‐0) was purchased from Cayman Chemical (Ann Arbor, MI, USA). Plasmids of human wild‐type STAT3 were encoded by pCMV6 vector with myc‐tag. The mutant SHP‐1 constructs (DN1 and D61A) have been generated to mimic the open‐form structure of SHP‐1 as previously described (Tai *et al*., 2014b). Antibodies for immunoblotting such as p‐VEGFR2, VEGFR2, p‐PDGFRβ, PDGFβ, p‐JAK1, JAK1, p‐JAK2, JAK2, p‐SRC, SRC, p‐STAT3, STAT3, and survivin were from Cell Signaling (Danvers, MA, USA). SHP‐1, cyclin D1, and Mcl‐1 antibodies were purchased from Abcam (Cambridge, MA, USA). Other antibodies such as poly (ADP‐ribose) polymerase (PARP) and cleaved caspase 3 were obtained from Cell Signaling Technology.

### 2.2 Cell culture and western blot analysis

The MCF10A human breast epithelial cell line, MCF7 luminal breast cancer cells, and TNBC (MDA‐MB‐231, MDA‐MB‐468, and HCC‐1937) cell lines were obtained from American Type Culture Collection (Manassas, VA, USA). All breast cancer cells were maintained in Dulbecco's modified Eagle's medium supplemented with 10% fetal bovine serum, 0.1 mm nonessential amino acids, 2 mm l‐glutamine, 100 U·mL^−1^ penicillin G, 100 μg·mL^−1^ streptomycin sulfate, and 25 μg·mL^−1^ amphotericin B in a 37 °C humidified incubator and an atmosphere of 5% CO_2_ in air. Lysates of breast cancer cells treated with drugs at the indicated doses and times were prepared for immunoblotting of p‐STAT3, STAT3, and other cells. Western blot analysis was performed as previously reported (Lehmann *et al*., 2015; Liu *et al*., 2013).

### 2.3 DNA fragmentation and apoptosis analysis

Cytoplasmic histone‐associated DNA fragments were determined as a measurement of apoptotic cells by the Cell Death Detection ELISAPLUS Kit (Roche, Indianapolis, IN, USA) according to the manufacturer's instructions. Drug‐induced apoptotic cell death was assessed using sub‐G1 analysis of propidium iodide‐stained cells by flow cytometry and western blot analysis of PARP cleavage.

### 2.4 Real‐time quantitative PCR

Total RNA was extracted from cultured cells using TRIzol reagent (Invitrogen), and real‐time quantitative PCR was performed in a LightCycler 480II instrument (Roche Diagnostics) using a LightCycler 480 SYBR Green I Master Kit (Roche Diagnostics), using specific primers for human cyclin D1 (forward primer, 5′‐GGATGCTGGAGGTCTGCGA‐3′; reverse primer, 5′‐AGAGGCCACGAACATGCAAG‐3′; 146 bp), human Mcl‐1 (forward primer, 5′‐GGTGCCTTTGTGGCTAAACA‐3′; reverse primer, 5′‐ACCCATCCCAGCCTCTTTGT‐3′; 133 bp), human survivin (forward primer, 5′‐AGAACTGGCCCTTCTTGGAGG‐3′; reverse primer, 5′‐CTTTTTATGTTCCTCTATGGGGTC‐3′; 170 bp), and the glyceraldehyde‐3‐phosphate dehydrogenase (GAPDH) gene was chosen as an internal control (forward primer, 5′‐CGACCACTTTGTCAAGCTCA‐3′; reverse primer, 5′‐AGGGGTCTACAT GGCAACTG‐3′; 228 bp).

### 2.5 Gene knockdown using small interfering RNA

Smart‐pool small interfering RNAs (siRNAs) including the control (D‐001810‐10) and SHP‐1 (PTPN6, L‐009778‐00‐0005) were purchased from Dharmacon (Chicago, IL, USA). The knockdown procedure was as described previously. Cells were transfected with siRNA (final concentration of 100 nm) in six‐well plates using the liposome transfection reagent Lipofectamine 2000 (Invitrogen) according to the manufacturer's instructions. After 72 h, the medium was replaced and the breast cancer cells were incubated with nintedanib, harvested, and separated for western blot analysis and apoptosis analysis by flow cytometry.

### 2.6 *In vitro* STAT3 Activity Assay

A Cignal Stat3 Reporter Kit (SABiosciences, Valencia, CA, USA) was used to measure the *in vitro* Stat3 activity. Cells were seeded in a 96‐well plate and transfected with reference pCMV‐Renilla luciferase plasmid with a plasmid driven by the promoter region containing STAT3‐specific binding sites and the constitutively expressing Renilla construct encodes the Renilla luciferase reporter gene and acts as an internal control for normalizing transfection efficiencies. After incubation for 48 h, the cells were treated with SC‐60 for 6 h and lysed with passive buffer. The lysates were transferred to a glass tube, and promoter activity was determined by Dual Luciferase Reporter Assay System (Promega, Madison, WI, USA) according to the manufacturer's instructions. Luciferase activities were measured on a GloMax 20/20 Luminometer (Promega). All the luciferase activities of samples were normalized with cells treated with DMSO.

### 2.7 SHP‐1 phosphatase activity

A RediPlate 96 EnzChek Tyrosine Phosphatase Assay Kit (R‐22067) was used for SHP‐1 activity assay (Molecular Probes, Carlsbad, CA, USA). The method was as described previously (Liu *et al*., 2013). Briefly, the protein extracts of breast cancer cells were incubated with anti‐SHP‐1 antibody in immunoprecipitation buffer overnight. Then, the Protein G‐Sepharose 4 Fast Flow (GE Healthcare, Piscataway, NJ, USA) was added to each sample followed by incubation for three hours at 4 °C with rotation and then assayed for phosphatase activity.

### 2.8 Xenograft tumor growth

The animal experiments were approved by the Institutional Animal Care and Use Committee of Taipei Veterans General Hospital. All experimental procedures using mice were performed in accordance with protocols approved by the Institutional Animal Care and Use Committee of Taipei Veterans General Hospital. Female NCr athymic nude mice (5–7 weeks of age) were obtained from the National Laboratory Animal Center (Taipei, Taiwan, Republic of China). The mice were housed in groups and maintained in a specific pathogen‐free environment. Each mouse was inoculated subcutaneously in the dorsal flank under isoflurane anesthesia with 2 × 10^6^ breast cancer cells suspended in 0.1 mL of serum‐free medium containing 50% Matrigel (BD Biosciences, San Jose, CA, USA). Tumors were measured using calipers, and their volumes were calculated using a standard formula: width^2 ^× length × 0.52. When tumors reached around 100 mm^3^, mice were administered SC‐60 (20 mg·kg^−1^ oral) three times a week. Controls received vehicle (50% (v/v) propylene glycol and 50% (v/v) Solutol^®^ HS‐15). Upon termination of treatment, mice were sacrificed and xenografted tumors were harvested and assayed for subsequent experiments.

### 2.9 Statistical analysis

Data are expressed as mean ± SD or SE. Statistical comparisons were based on nonparametric tests, and statistical significance was defined as a *P* value of less than 0.05. For survival analysis, progression‐free survival curves of patients were generated using the Kaplan–Meier method and compared by performing a log‐rank test. All statistical analyses were carried out using spss for Windows software, version 12.0 (SPSS, Chicago, IL, USA).

## 3 Results

### 3.1 SC‐60 shows a growth inhibitory effect in human breast cancer cells

**Figure 1:**
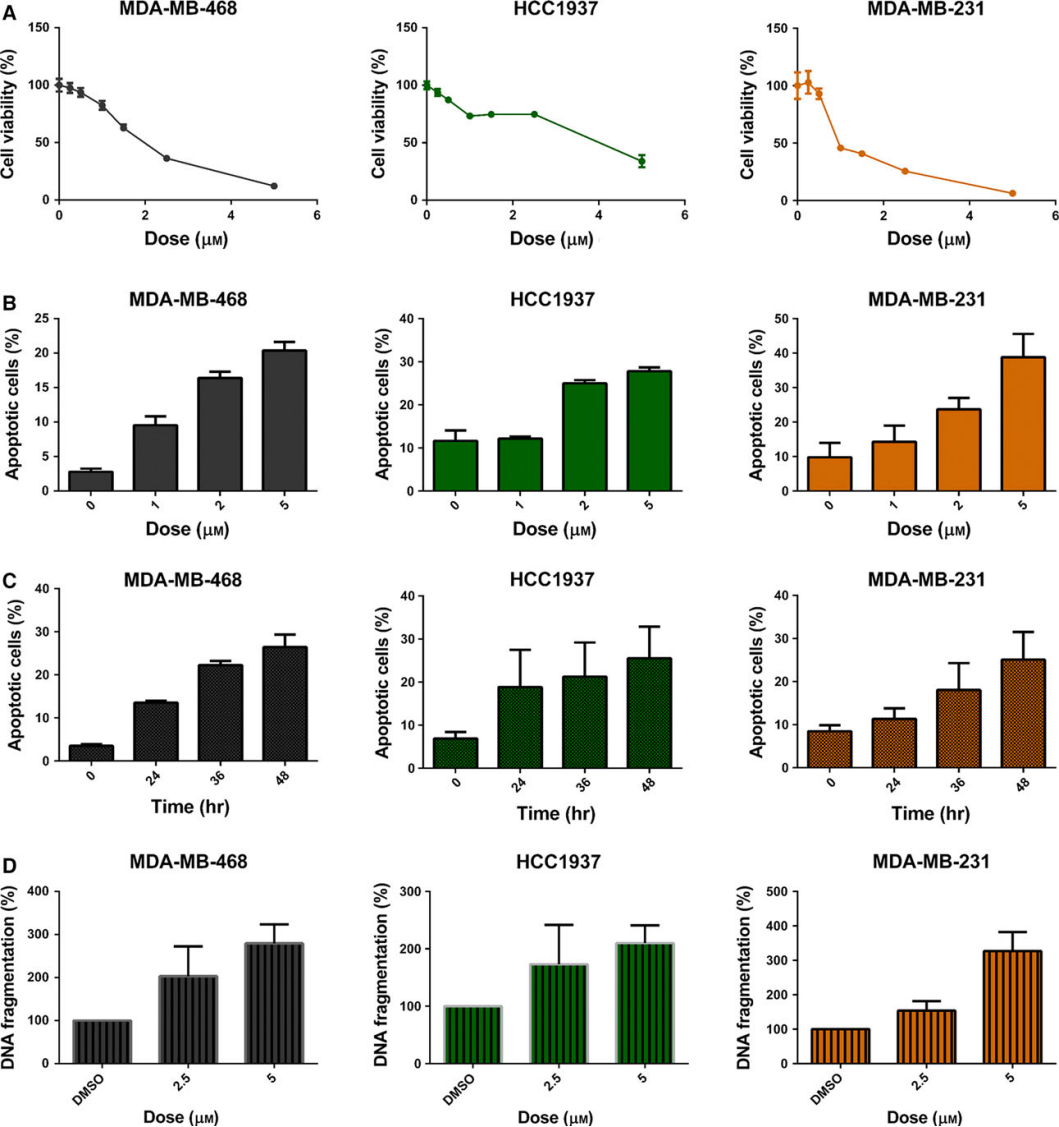
SC‐60 shows antiproliferative effects in human triple‐negative breast cancer cells. (A) Cells were exposed to SC‐60 at the indicated doses for 72 h, and cell viability was assessed by MTT assay. (B,C) Flow cytometry assay revealed that SC‐60 treatment resulted in increased percentage of apoptotic cells in the aforementioned cell lines in a dose (B)‐ and time (C)‐dependent manner. (D) SC‐60 treatment led to increased DNA fragmentation in TNBC cells in a dose‐dependent manner. Means of at least three independent experiments performed in triplicate are shown. Data are shown as mean ± SD.

To evaluate the efficacy of SC‐60 on human TNBC cells, we treated three human TNBC lines with different doses of SC‐60 for 72 h, and the cell viability was analyzed by MTT assay. As shown in Fig. 1A, SC‐60 dose dependently inhibited cell growth of MDA‐MB‐468, HCC1937, and MDA‐MB‐231 cells. In addition, flow cytometry assay revealed that SC‐60 treatment resulted in increases in the percentage of apoptotic cells in the aforementioned cell lines in a dose (Fig. 1B)‐ and time (Fig. 1C)‐dependent manner. In accordance with the above results, SC‐60 treatment also led to increased DNA fragmentation in human breast cancer cells in a dose‐dependent manner (Fig. 1D). To further test the cytotoxic effect of SC‐60 on normal MCF‐10A human breast epithelial cells and MCF‐7 luminal breast cancer cells, we also performed the MTT assay and flow cytometry analysis. As shown in Fig. S2A, SC‐60 showed antiproliferative effect on MCF‐10A as well as MCF‐7 cells. In addition, SC‐60 induced mild apoptosis‐inducing effect on MCF‐10A at 5 μm and MCF‐7 at 2 and 5 μm compared to that of TNBC cell lines (Figs 1B and S2B). Our results showed that SC‐60 had more cytotoxic effects on breast cancer cells than on normal breast MCF‐10A cells.

### 3.2 SC‐60 enhanced cell apoptosis through p‐STAT3 inhibition

**Figure 2:**
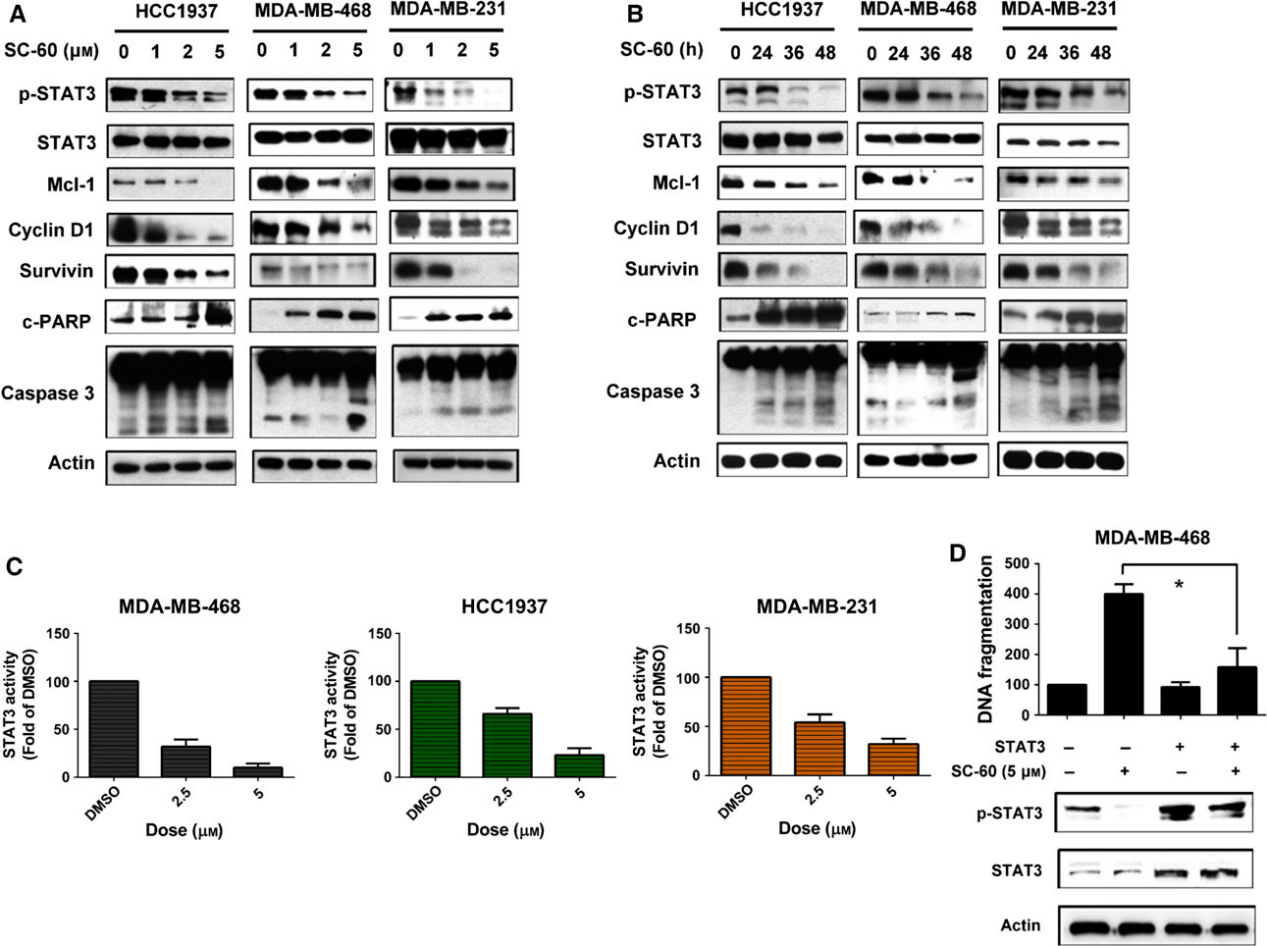
SC‐60 enhances apoptosis and reduces p‐STAT3 signaling in TNBC cells. (A,B) The effects of SC‐60 on p‐STAT3 and its downstream molecules (Mcl‐1, cyclin D1, and survivin) were analyzed by western blot. Cells were treated with SC‐60 at the (A) indicated doses for 48 h or (B) treated with SC‐60 (5 μm) at the indicated times. (C) Cells were transfected with inducible STAT3‐responsive firefly luciferase construct and constitutively expressing Renilla luciferase construct as internal control for 48 h; then, the cells were treated with SC‐60 at indicated dose for 6 h. The activities of STAT3 were measured by dual luciferase assay. The activities of STAT3 of tested samples were normalized with cells treated with DMSO. (D) Overexpression of STAT3 reversed the apoptotic effect of SC‐60. MDA‐MB‐468 cells were transfected with STAT3‐expressing vector with myc‐tag for 24 h and then treated with DMSO or SC‐60 at 5 μm for another 24 h. DNA fragmentation was measured, and the effect on p‐STAT3 was analyzed by western blot. Means of at least three independent experiments performed in triplicate are shown. *P <* *0.05. Data are shown as mean ± SD.

To investigate the mechanisms by which SC‐60 inhibited cell growth and induced cell apoptosis in TNBC, we analyzed the protein expressions of p‐STAT3 and its downstream proteins that have been demonstrated to be involved in cancer cell proliferation and survival. We found that SC‐60 inhibited the expression of p‐STAT3 and its downstream effectors including Mcl‐1, cyclin D1, and survivin in TNBC cells in a dose‐ and time‐dependent manner. Because STAT3 has been reported as a transcription factor, therefore, we also checked whether SC‐60 affected the mRNA levels of its downstream molecules. As shown in Fig. S3, SC‐60 indeed decreased the mRNA levels of STAT3 downstream target genes, cyclin D1, Mcl‐1, and survivin in MDA‐MB‐231 cells. Furthermore, the levels of apoptosis‐related proteins were also activated by SC‐60 in a dose‐ and time‐dependent manner in TNBC cells (Fig. 2A,B). To analyze whether the activity of STAT3 is affected by SC‐60, luciferase assays were performed using the Cignal STAT3 Reporter Assay Kit. TNBC cells were transfected with reference pCMV‐Renilla luciferase plasmid with a plasmid driven by the promoter region containing STAT3‐specific binding sites and treated with SC‐60. Results showed that SC‐60 dose dependently decreased the activity of STAT3 in TNBC cells (Fig. 2C). To further validate the role of STAT3 in SC‐60‐induced apoptosis in TNBC cells, we established stable STAT3‐overexpressing MDA‐MB‐468 cells. As shown in Fig. 2D, constitutively expressing STAT3 reversed SC‐60‐mediated p‐STAT3 inhibition and suppressed the apoptotic effect of SC‐60 on MDA‐MB‐468 cells, indicating that STAT3 mediates SC‐60‐induced apoptosis in TNBC cells.

As there are other molecular events that typically modulate STAT3 activity, such as VEGFR2, PDGFRβ, JAK1, JAK2, and ERK1/2 (Schreiner *et al*., 2002; Tian and An, 2004; Yu *et al*., 2014), we also examined the effects of SC‐60 on these molecules and found that SC‐60 did not affect VEGFR2, PDGFRβ, JAK1, JAK2, and ERK1/2 (Fig. S4).

### 3.3 SHP‐1 mediates effects of SC‐60 on p‐STAT3 inhibition in TNBC cells

**Figure 3:**
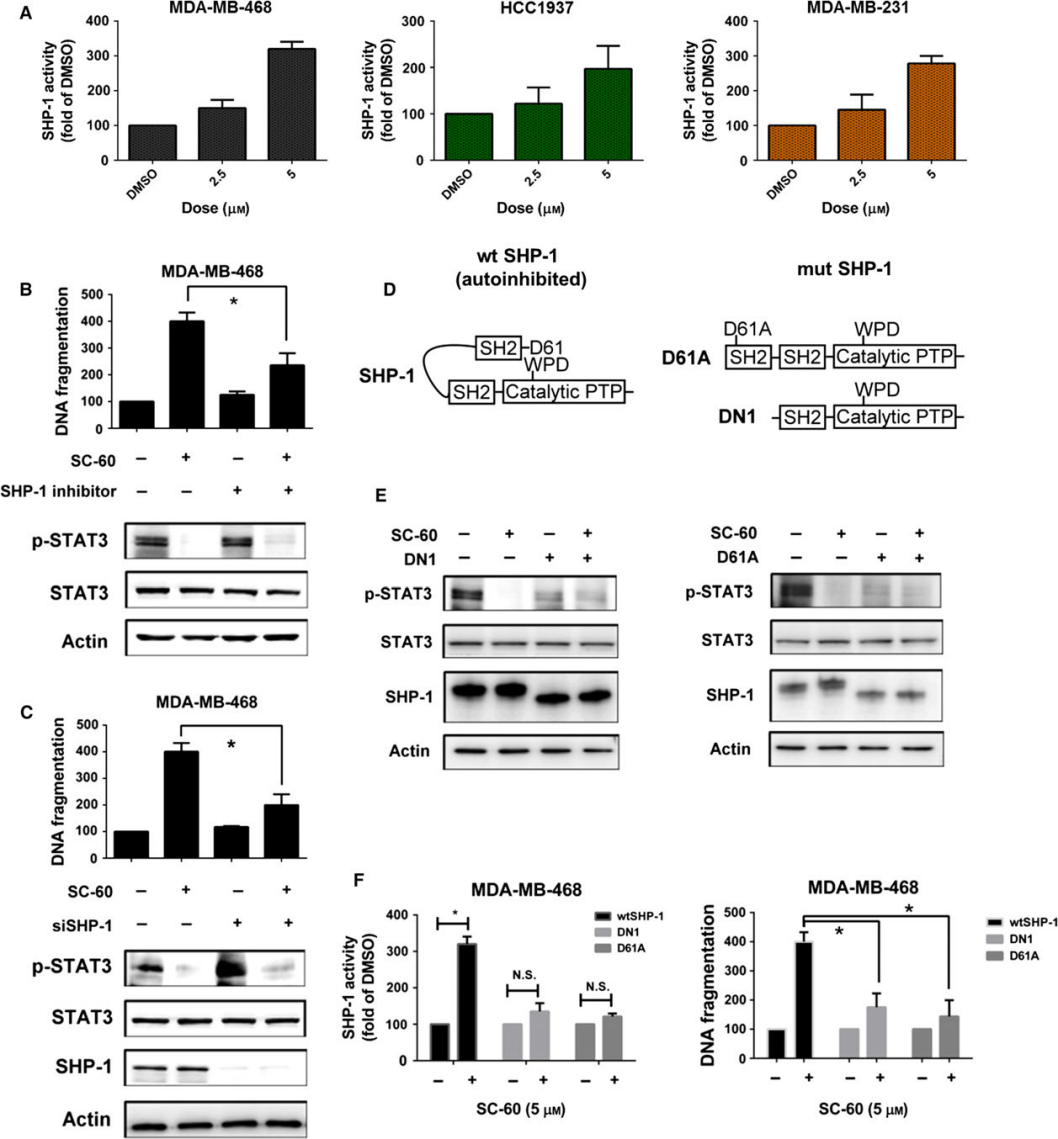
SC‐60 induces cell apoptosis by SHP‐1/p‐STAT3 signaling in TNBC cells. (A) The activities of SHP‐1 were measured at the indicated doses for 48 h in MDA‐MB‐468, HCC1937, and MDA‐MB‐231 TNBC cell lines. (B) The protective effects of SHP‐1 inhibitor on SC‐60‐induced apoptosis in MDA‐MB‐468 cells. Cells were pretreated with 50 μm SHP‐1 inhibitor (PTP inhibitor III) for 1 h and then treated with SC‐60 at 5 μm for 36 h. DNA fragmentation was determined by the Cell Death Detection ELISAPLUS Kit. (C) Knockdown of SHP‐1 reversed the biological effects of SC‐60 on apoptosis (left) and p‐STAT3 inhibition (right). MDA‐MB‐468 cells were transfected with control siRNA (scrambled) or SHP‐1 siRNA for 24 h and then treated with SC‐60 at 5 μm for another 24 h. The protein levels of p‐STAT3, STAT3, SHP‐1, and actin (as loading control) were analyzed by western blot, and DNA fragmentation was measured by Cell Death Detection ELISAPLUS Kit. (D) Schematic representation of wild‐type SHP‐1 (autoinhibited), deletion and single mutants of SHP‐1 (mimic activated SHP‐1). (E,F) MDA‐MB‐468 cells were transfected with mutant SHP‐1 (DN1 or D61A) for 24 h and then treated with SC‐60 at 5 μm for another 24 h. (E) The protein levels of p‐STAT3, STAT3, SHP‐1, and actin (as loading control) were analyzed by western blot. (F) The SHP‐1 activity was assessed by SHP‐1 phosphatase activity (left) and DNA fragmentation (right) was measured by Cell Death Detection ELISAPLUS Kit as described in 2. Means of at least three independent experiments performed in triplicate are shown. *P <* *0.05. Data are shown as mean ± SD.

To further determine the mechanism by which SC‐60 inhibits STAT3 phosphorylation, we analyzed the activity of its phosphatase, SHP‐1, in SC‐60‐treated cells. As shown in Fig. 3A, SC‐60 increased SHP‐1 activity significantly in three TNBC cell lines. We then added a specific SHP‐1 inhibitor, an α‐haloacetophenone derivative PTP inhibitor III that acts as a covalent inhibitor of PTPs and binds the catalytic domain of SHP‐1 (Arabaci *et al*., 1999), in MDA‐MB‐468 cells and found that it significantly reduced SC‐60‐induced downregulation of p‐STAT3 and apoptosis (Fig. 3B). Similarly, silencing SHP‐1 with small interfering RNA (siRNA) in MDA‐MB‐468 cells inhibited the effects of SC‐60 on p‐STAT3 inhibition and cell apoptosis induction (Figs 3C and S5). Previous studies have indicated that the autoinhibitory structure from the N‐SH2 domain to PTP domain is the major regulator of SHP‐1 activity (Wu *et al*., 2003; Yang *et al*., 1998, 2003). The mutant SHP‐1 constructs (DN1 and D61A) shown in Fig. 3D have been generated to mimic the open‐form structure of SHP‐1 (Tai *et al*., 2014b), which showed higher SHP‐1 activities than wild‐type SHP‐1. Therefore, these two mutants would show more potent inhibition on p‐STAT3 expression due to higher SHP‐1 activities (Fig. 3E,F, left). Moreover, it is expected that SC‐60 would interfere the open/close structures of SHP‐1, thereby increasing the SHP‐1 activity. Indeed, when the SHP‐1 was maintained as open structure (DN1 and D61A mutants), the effects of SC‐60 on p‐STAT3 inhibition (Fig. 3E) and apoptosis were reduced (Fig. 3F, right).

### 3.4 SC‐60 inhibits MDA‐MB‐468 tumor growth *in vivo*

**Figure 4:**
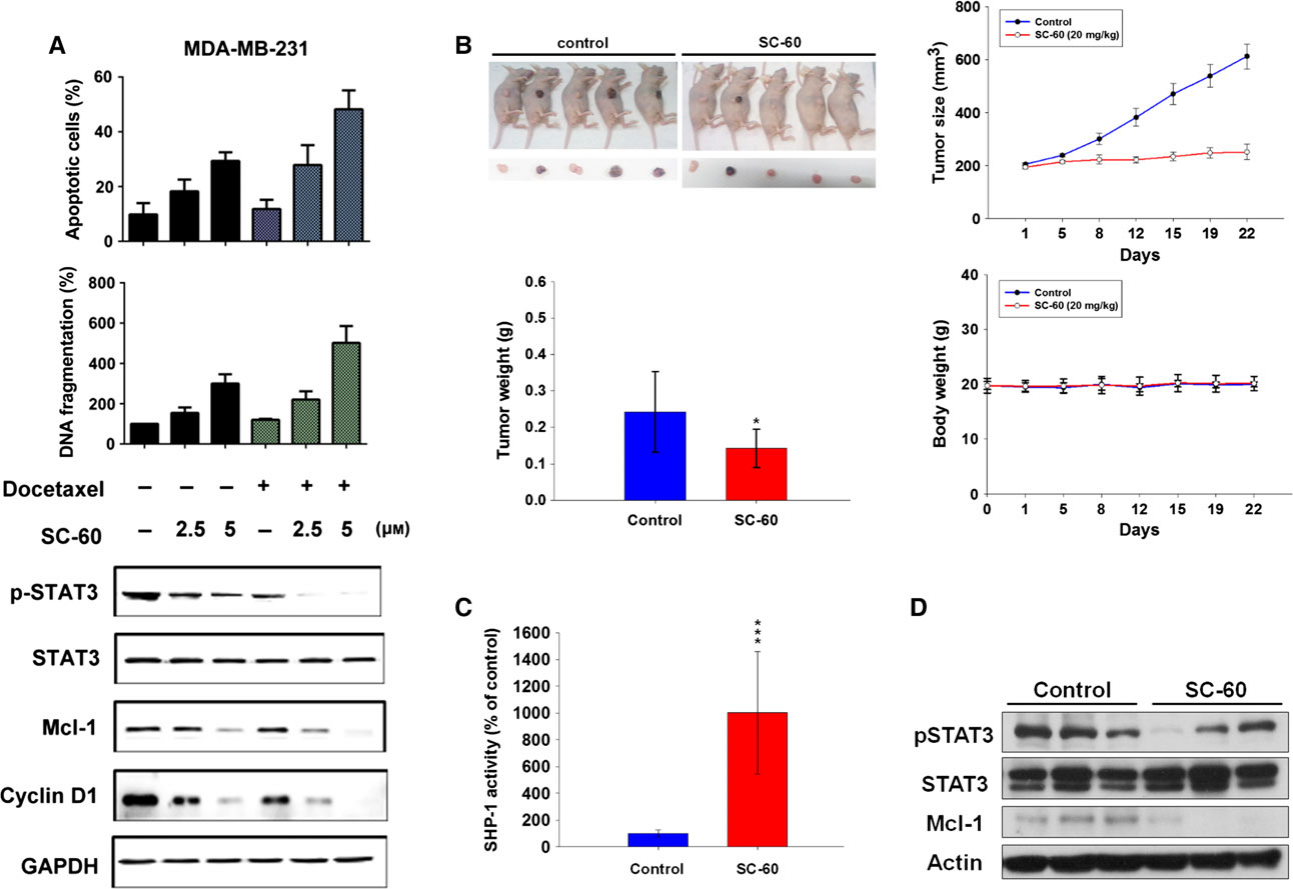
Combination of SC‐60 and docetaxel increases cell apoptosis by reducing p‐STAT3, and SC‐60 diminishes xenograft tumor growth of TNBC cells. (A) Cells were treated with docetaxel (0.1 μm) for 24 h, then treated with SC‐60 at the indicated doses (0, 2.5, and 5 μm) for another 24 h. Effect of docetaxel/SC‐60 combination on cell apoptosis (upper), DNA fragmentation (middle), p‐STAT3 and its downstream signaling (lower) were measured. Means of at least three independent experiments performed in triplicate are shown. Data are shown as mean ± SD. (B–D) MDA‐MB‐468‐bearing mice were treated with vehicle or SC‐60 orally at 20 mg·kg^−1^ three times a week. (B) Mice images (upper left), growth curves (lower left), tumor weight (upper right), body weight (lower right), (C) SHP‐1 activity, and (D) western blot analysis of p‐STAT3, STAT3, and Mcl‐1 were measured. Data of growth curve (*n* = 5) are shown as mean ± SE. Data of tumor weight, body weight, and SHP‐1 activity (*n* = 5) are shown as mean ± SD. *P <* *0.05. ***P <* *0.001.

To investigate the clinical therapeutic options, we combined SC‐60 with docetaxel in MDA‐MB‐231 cells. This combination increased apoptosis (Fig. 4A, upper), DNA fragmentation (Fig. 4A, middle), and strongly reduced STAT3 phosphorylation (Fig. 4A, lower) in comparison with SC‐60 treatment alone. These results suggest that the combination of a lower dose of SC‐60 with docetaxel provides new therapeutic option in the treatment of patients with TNBC. To determine whether the effect of SC‐60 on TNBC cells is potentially clinically relevant, we tested the *in vivo* effect of SC‐60 on tumor growth in a xenograft mouse model. We found that SC‐60 treatment showed reduced tumor weight and suppressed tumor growth (Figs 4B and S6), increased SHP‐1 activity (Fig. 4C), and decreased p‐STAT3 and Mcl‐1 expressions in xenografted tumors (Figs 4D and S6). These results indicated that SC‐60 inhibited tumor growth through STAT3 inactivation.

## 4 Discussion

In the current study, we demonstrated the *in vitro* effectiveness of SC‐60 in three TNBC cell lines MDA‐MB‐468, HCC1937, and MDA‐MB‐231, and its *in vivo* antitumor efficacy in a MDA‐MB‐468 xenograft mouse model. We confirmed that SC‐60 exhibits its efficacy via the SHP‐1/p‐STAT3 pathway (Fig. 3). Moreover, the potential combination with docetaxel was shown by enhanced apoptosis *in vitro* (Fig. 4). Our study further reinforces the notion that targeting p‐STAT3 by enhancing SHP‐1 activity may be a useful anticancer therapeutic approach, and also provides a new chemical entity with pharmacological potential.

**Figure 5:**
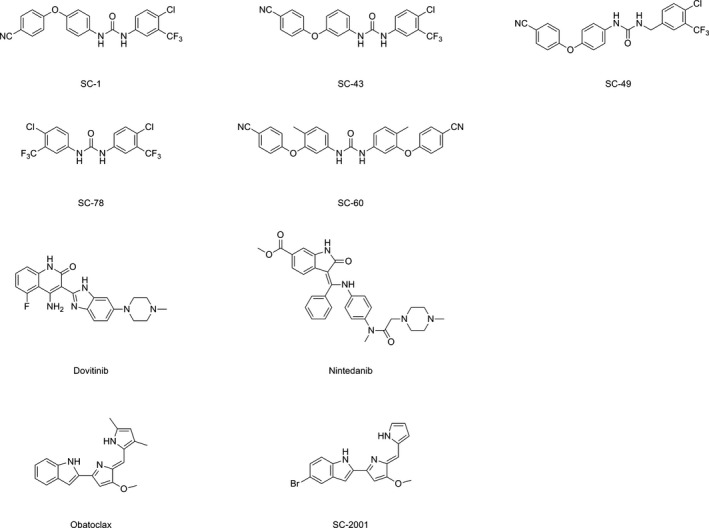
Chemical structures of compounds or drugs that have been reported to enhance SHP‐1 activity.

At present, there are no clinically approved STAT3‐targeted agents. One possible hindrance may be that direct inhibition of STAT3 dimerization requires total inhibition of STAT3 molecules in the cell, which may need high drug concentrations (Chai *et al*., 2016; Furtek *et al*., 2016). In contrast, kinase inhibitors or enzyme activators may be feasible approaches (Zhang *et al*., 2015; Zhong *et al*., 2015). JAK kinase inhibitors, the so‐called jakinibs, were originally designated as therapy for myeloproliferative diseases (Kontzias *et al*., 2012). Recently, the therapeutic implication of these jakinibs has moved toward cancer and autoimmune diseases such as rheumatoid arthritis (Kontzias *et al*., 2012). Indeed, the JAK inhibitor ruxolitinib is currently in clinical trials for solid cancers (Hurwitz *et al*., 2014) and is closer to clinical approval for cancer therapy compared with other STAT3‐targeting agents. As mentioned earlier, agents aiming to inhibit STAT3 phosphorylation other than JAK inhibitors are also an interesting field of drug development. Previously, we demonstrated that a SHP‐1 enhancer, SC‐43, is effective for TNBC cells (Liu *et al*., 2013). A number of compounds or drugs have been reported to be capable of enhancing SHP‐1 activity (Fig. 5). Sorafenib analogues with a urea‐based structure (SC agents such as SC‐1, SC‐49, SC‐43, SC‐60, and SC‐78) have been tested as SHP‐1 enhancers for anticancer activity in preclinical settings (Chen *et al*., 2012c; Fan *et al*., 2014; Liu *et al*., 2013; Su *et al*., 2016; Tai *et al*., 2011). Among these agents, SC‐60 has been found to form the hydrogen bonding with N280 of the PTP domain through a docking model (Fan *et al*., 2014). Another class of SHP‐1 enhancer is obatoclax analogue, SC‐2001, increasing SHP‐1 expression through transcription factor RFX‐1 (Chen *et al*., 2012b; Liu *et al*., 2014; Su *et al*., 2012, 2014a,b). The core structure of obatoclax and SC‐2001 includes a pyrrole and indole ring. Nintedanib has been found to elevate SHP‐1 activity through interacting with SHP‐1 at Glu524 through hydrogen bonding in a docking model (Tai *et al*., 2014c). Although many SHP‐1 activators have been identified (Chen *et al*., 2012a,b,c; Liu *et al*., 2014; Su *et al*., 2014a,b, 2016; Tai *et al*., 2012), the structure and activity relationship still needs to be further investigated.

It is clear that many solid cancers have aberrant and activated JAK/STAT signaling (Roxburgh and McMillan, 2016; Sansone and Bromberg, 2012). Despite STAT3 signaling not being specific for TNBC, targeting STAT3 activation in TNBC is feasible for several reasons: First, currently there are no single pathways specific for therapy for all subtypes of TNBC (Mayer *et al*., 2014). Given the heterogeneity of TNBC revealed by molecular profiling (Lehmann *et al*., 2011; Mayer *et al*., 2014), the general applicability of STAT3 signaling in cancer cells provides an alternative strategy to traditional chemotherapy. Moreover, we also demonstrated the potential combination of chemotherapeutics with SHP‐1 agonists (Fig. 4), suggesting the possibility of combining p‐STAT3 inhibitors with chemotherapy in the future. Furthermore, we recently discovered a VEGF‐A‐dependent autocrine/paracrine loop in TNBC which could be disrupted by SHP‐1 enhancers, suggesting that the SHP‐1/p‐STAT3/VEGF‐A axis is a potential therapeutic target for metastatic TNBC (Su *et al*., 2016). Interestingly, our results showed that SC‐60 had more cytotoxic effects on breast cancer cells than on normal breast MCF10A cells, and it seemed that TNBC cells might be more sensitive than hormone receptor‐positive MCF‐7 cells in terms of apoptotic effects (Figs 1 and S2). Estrogen is a growth factor for hormone receptor‐positive breast cancer cells and may contribute to chemoresistance (Jiang *et al*., 2012). Moreover, estrogen receptor (ERα) can bind to STAT3 and JAK2, resulting in enhanced JAK2 activity upstream of STAT3 in response to stimulation which might lead to an increased (ERα)‐dependent cell viability (Binai *et al*., 2010). However, it remains speculative and more studies are necessary to address possible mechanisms for the differential apoptosis sensitivities among TNBC and hormone receptor‐positive breast cancer cell lines.

Notwithstanding, the prognostic role of p‐STAT3 in cancer patient outcome seems to be conflicting among various solid cancers (Thomas *et al*., 2015). A recent review summarizing studies on the relationship between JAK/STAT activation and prognosis suggested that most studies have utilized immunohistochemistry to determine p‐STAT3 signaling (Thomas *et al*., 2015). In several cancers such as prostate, non‐small‐cell lung cancers, cervical cancers, renal cell carcinoma, and glioblastoma, activation of STAT3 or STAT5 is associated with a worse prognosis; conversely, STAT3 is associated with favorable prognosis in breast cancer and in some studies in colorectal cancer and head and neck squamous cell carcinoma (Thomas *et al*., 2015). This difference in prognosis prediction may be partly due to different tumor biology among cancers, and by the various regulatory mechanisms upstream of p‐STAT3 signaling, for example, endogenous negative regulators such as the suppressor of cytokine signaling family, protein inhibitor of activated STAT (PIAS) proteins and the PTP family, or post‐translational modifications (Chai *et al*., 2016).

In this study, we showed that SC‐60 acts as a SHP‐1 agonist. SC‐60 increased SHP‐1 activity, thereby decreased p‐STAT3, and subsequently decreased cyclin D1 expression. However, SC‐60 seemed to affect cyclin D1 expression more prominently compared to p‐STAT3 expression (Fig. 2). Cyclin D1 can be regulated by numerous effectors, such as STAT3 (Leslie *et al*., 2006), ERK1/2 (Balmanno and Cook, 1999), and SRC/PI3K/AKT (Xing *et al*., 2008) pathways. This may indicate that p‐STAT3 is not the only effector in SC‐60‐mediated cyclin D1 suppression. There might be other STAT3‐independent effects of SC‐60 on cyclin D1. Moreover, previous studies have shown that SHP‐1 also regulates MAPK/ERK pathway by dephosphorylating ERK (Cai *et al*., 2006) and that lack of SHP‐1 prevents phosphorylation of the active site (Tyr416) on Src (Okenwa *et al*., 2013). In contrast, our data showed that SC‐60 did not significantly alter the phosphorylation of ERK1/2 (Fig. S4), whereas the phosphorylation of Src on Try416 was found to be mildly decreased with SC‐60 treatment (Fig. S5). However, why SC‐60 (as a SHP‐1 agonist) could affect p‐Src (Try416) is not clearly understood; we suggest that SC‐60 may exert other than SHP‐1 agonist activity (off‐target effect) that decreases p‐Src (Try416) or may through other effectors to decrease the expression of cyclin D1. Nevertheless, further studies are necessary.

In summary, our study demonstrates the preclinical activity of a SHP‐1 agonist SC‐60 in TNBC, the therapeutic implication of targeting SHP‐1/p‐STAT3, and the potential combination of a SHP‐1 agonist with chemotherapeutic docetaxel as a feasible therapeutic strategy for TNBC.

## Author contributions

CYL, JCS, and TTH drafted the manuscript. CYL, JCS, TTH, CHL, KYL, WCT, HPY, and CTH conducted *in vitro* experiments. PYC, WLW, TTH, and JCS conducted animal experiments. CWS, LMT, and KFC helped in data interpretation and statistical analysis. CYL, JCS, TTH, PYC, CTH, and WLW prepared the figures. CWS and KFC designed and synthesized SC‐60. All authors made substantial contributions to the conception or design of the work. All authors have read, revised critically for intellectual content, and approved the final manuscript. All authors agreed with the accuracy and integrity of all parts of the work.

## Supporting information


**Fig. S1.** The chemical structure and solubility of SC‐60.
**Fig. S2.** Cytotoxicity effect of SC‐60 on MCF‐10A normal human breast epithelial cells and MCF‐7 luminal breast cancer cells.
**Fig. S3.** SC‐60 decreased the mRNA levels of STAT3 downstream target genes.
**Fig. S4.** SC‐60 had no obvious effects on VEGFR2, PDGFRβ, JAK1, JAK2 and ERK1/2 in MDA‐MB‐231 cells.
**Fig. S5.** The effects of SC‐60 on SHP1‐depleted MDA‐MB‐468 cells.
**Fig. S6.** SC‐60 diminishes xenograft tumor growth of MDA‐MB‐468 cells.
