# Supplementary material for: Sorafenib analogue SC‐60 induces apoptosis through the SHP‐1/STAT3 pathway and enhances docetaxel cytotoxicity in triple‐negative breast cancer cells
Source: Mol Oncol. 2017 Feb 7;11(3):266–79. doi: 10.1002/1878-0261.12033 (PMC5527447; doi:10.1002/1878-0261.12033)
Supplement: Supplementary file 1 — Fig. S1. The chemical structure and solubility of SC‐60. Fig. S2. Cytotoxicity effect of SC‐60 on MCF‐10A normal human breast epithelial cells and MCF‐7 luminal breast cancer cells. Fig. S3. SC‐60 decreased the mRNA levels of STAT3 downstream target genes. Fig. S4. SC‐60 had no obvious effects on VEGFR2, PDGFRβ, JAK1, JAK2 and ERK1/2 in MDA‐MB‐231 cells. Fig. S5. The effects of SC‐60 on SHP1‐depleted MDA‐MB‐468 cells. Fig. S6. SC‐60 diminishes xenograft tumor growth of MDA‐MB‐468 cells. [file MOL2-11-266-s001.pdf]

Supplementary figures and figure legends

Supplementary Figure S1

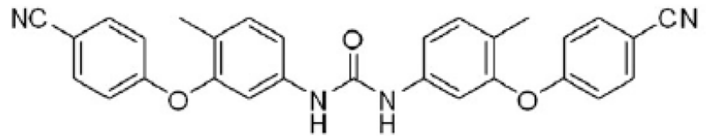

SC-60

Molecular weight: 473

Solubility: 1.49 mg/ml in ethanol
1.96 mg/ml in acetonitrile
> 2 mg/ml in 12.5% EtOH +12.5% Cremophor EL + 75% H2O (v:v:v)
> 2 mg/ml in 50% Propylene glycol + 50% Solutol HS 15 (v:v)
> 2 mg/ml in 50% Propylene glycol + 50% Vitamin E TPGS (v:v)

Source: Synthesized by Hinova Pharmaceuticals, Inc.

Figure S1－The chemical structure and solubility of SC-60. Molecular weight and solvents that are able to dissolve SC-60 are listed.

Supplementary Figure S2

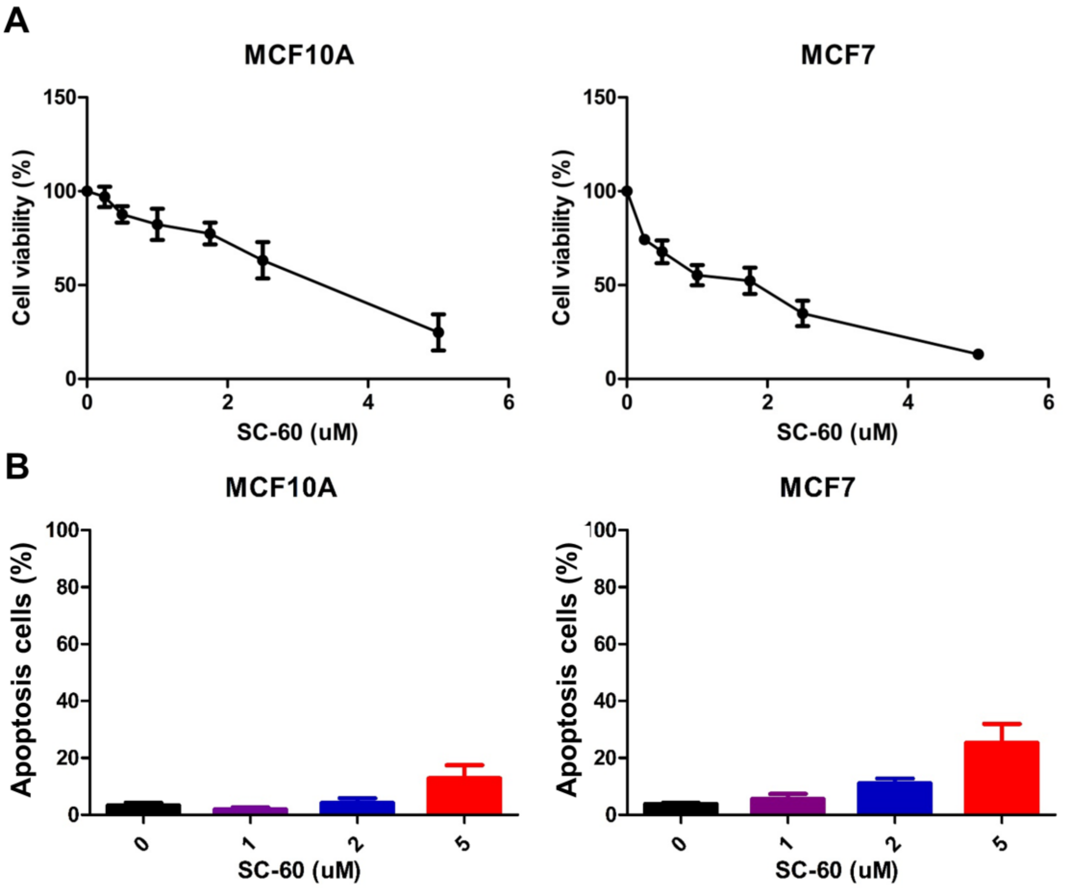

Figure S2 — Cytotoxicity effect of SC-60 on MCF-10A normal human breast epithelial cells and MCF-7 luminal breast cancer cells. (A) MCF-7 and MCF-10A cells were exposed to SC-60 at the indicated doses (0, 0.25, 0.5, 1, 1.75, 2.5, 5 μM) for 72 h and cell viability was assessed by MTT assay. (B) MCF-7 and MCF-10A cells were exposed to SC-60 at the indicated doses (0, 1, 2, 5 μM) for 48 h and the apoptotic cells were determined by flow cytometry. Means of at least three independent experiments performed in triplicate are shown. Data are shown as mean ± SD.

Supplementary Figure S3

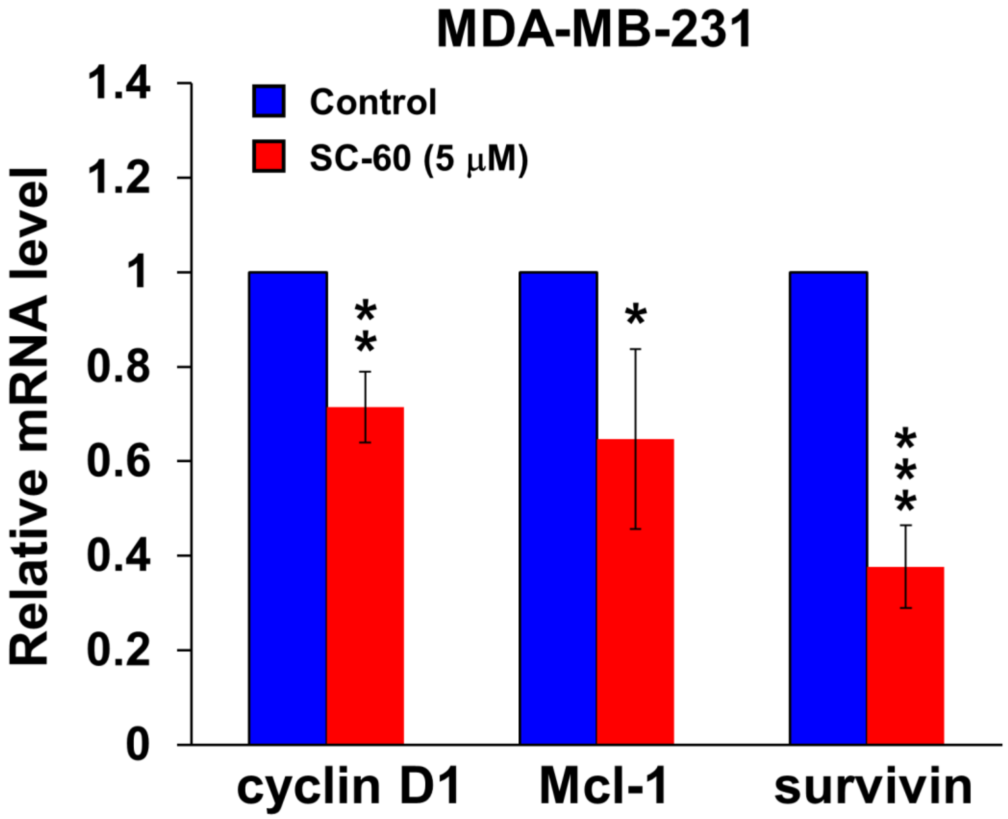

Figure S3－SC-60 decreased the mRNA levels of STAT3 downstream target genes.

Cells were treated with SC-60 (5 μM) for 12 h and the mRNA levels of cyclin D1, Mcl-1 and survivin were analyzed by real-time quantitative PCR. Data are shown as mean ± SD. n=3; *, $P < 0.05$; **, $P < 0.01$; ***, $P < 0.001$.

Supplementary Figure S4

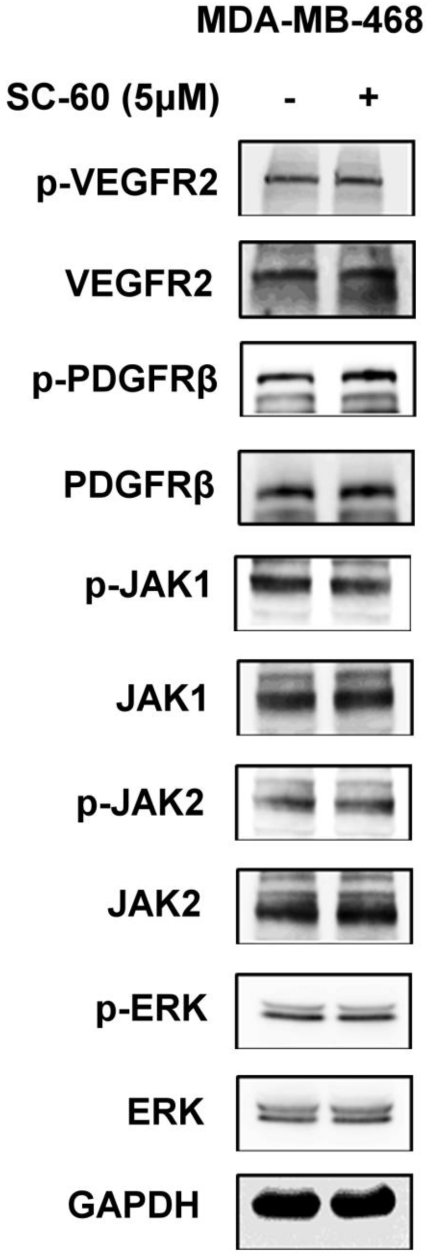

Figure S4－SC-60 had no obvious effects on VEGFR2, PDGFRβ, JAK1, JAK2 and ERK1/2 in MDA-MB-231 cells. MDA-MB-231 cells were treated with SC-60 (5 μM) or DMSO for 48 h, and protein levels of VEGFR2, p-VEGFR, PDGFRβ, p-PDGFRβ, JAK1, p-JAK1, JAK2, p-JAK2, ERK1/2, p-ERK1/2 and GAPDH were analyzed by Western blot.

Supplementary Figure S5

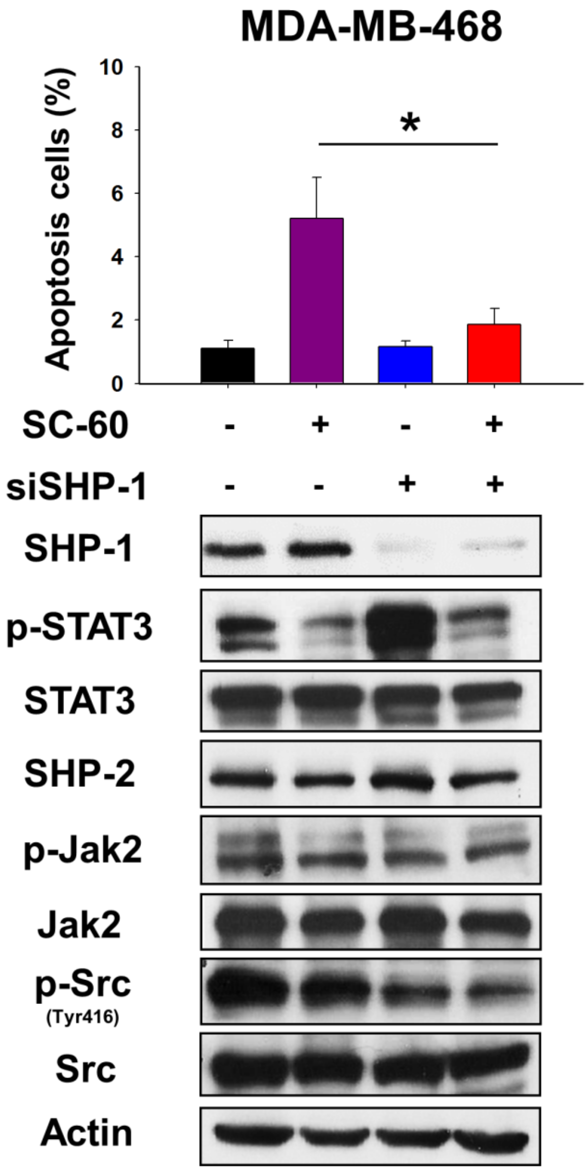

Figure S5—**The effects of SC-60 on SHP1-depleted MDA-MB-468 cells.** MDA-MB-468 cells were transfected with control siRNA (scrambled) or SHP-1 siRNA for 48 h then treated with SC-60 at 5 μM for another 18 h. The sub-G1 population was analyzed by flow cytometry. Protein expressions of SHP-1, SHP-2, p-STAT3, STAT3, p-JAK2, JAK2, p-Src, Src and actin (as loading control) were measured by Western blot analysis.

Supplementary Figure S6

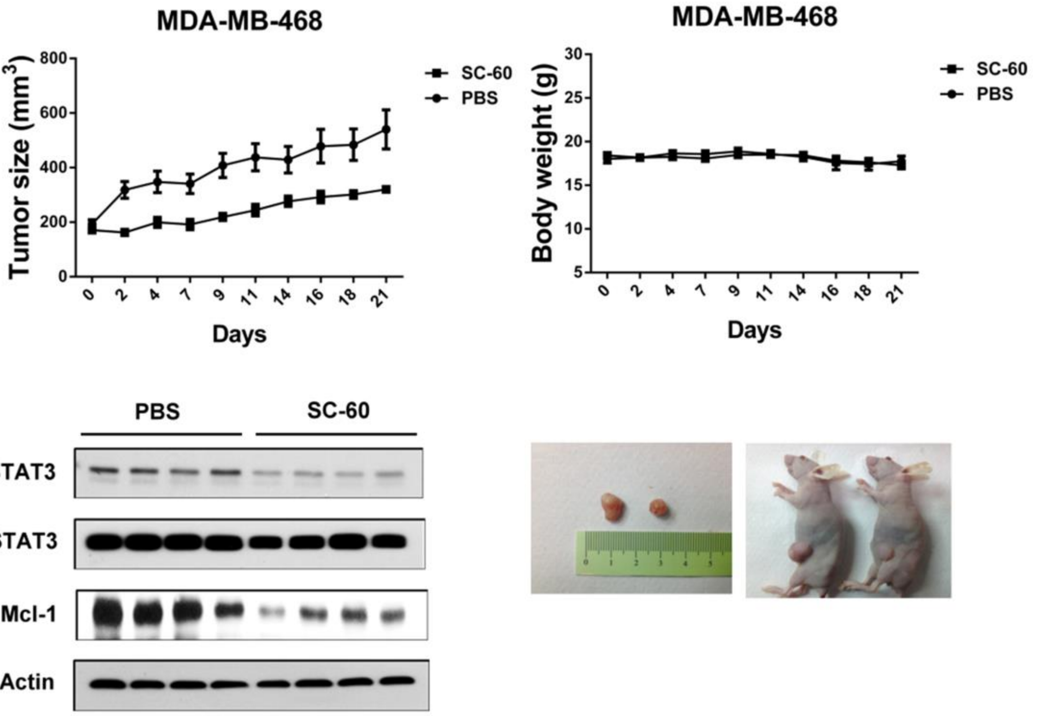

Figure S6 — SC-60 diminishes xenograft tumor growth of MDA-MB-468 cells. MDA-MB-468-bearing mice (n=6) were treated with vehicle (1x PBS) or SC-60 orally at 20 mg/kg three times a week. The growth curves (*upper left*), body weight (*upper right*), and Western blot analysis of p-STAT3, STAT3 and Mcl-1 (*lower left*) and mice images (*lower right*) were measured. Data are shown as mean ± SE.
